# Exploring associations between constipation, severity of neurofibromatosis type 1 and *NF1* mutational spectrum

**DOI:** 10.1038/s41598-021-87686-x

**Published:** 2021-04-28

**Authors:** Cecilie Ejerskov, Mette Gaustadnes, John R. Ostergaard, klaus Krogh, Kasper Thorsen, Anders D. Borglum, Annette Haagerup

**Affiliations:** 1 Centre for Rare Diseases, Department of Paediatrics and Adolescent Medicine, Aarhus University Hospital, Palle Juul-Jensens Boulevard 99, 8200 Aarhus N, Denmark; 2 Department of Clinical Medicine, Aarhus University, Aarhus, Denmark; 3 NIDO|Denmark, Research in Education and Health, Regional Hospital West Jutland, Gl. Landevej 61, 7400 Herning, Denmark; 4 Department of Molecular Medicine, Aarhus University Hospital, Brendstrupgårdsvej 21 A, 8200 Aarhus N, Denmark; 5 Department of Hepatology and Gastroenterology, Aarhus University Hospital, Palle Juul-Jensens Boulevard 99, 8000 Aarhus N, Denmark; 6 Department of Biomedicine, Aarhus University, Vennelyst Boulevard 4, 8000 Aarhus, Denmark; 7 Centre for Integrative Sequencing, iSEQ, Aarhus University, Aarhus, Denmark; 8 Lundbeck Foundation for Integrative Psychiatric Research, iPSYCH, Aarhus University, Aarhus, Denmark

**Keywords:** Diseases of the nervous system, Clinical genetics

## Abstract

Neurofibromatosis type 1 (NF1) is inherited in an autosomal dominant manner and is a rather common rare disease. Until recently, studies on gastrointestinal symptoms in patients with NF1 have been few and mostly described as case reports. In three previously published studies, the frequency of constipation in patients with NF1 has been found to be as high as 30%. In this study, associations between the frequency of constipation and NF1 disease severity and *NF1* mutational spectrum were investigated. Among 277 patients with NF1, 49 had constipation. The highest rate of constipation was found among patients with a high perception of NF1 illness burden, and patients with constipation had a significantly higher NF1 illness burden when comparing the “not bothered” and the “very bothered” (*p* = 0.013). We found no significant association between constipation and the remaining measures on severity of NF1, nor between constipation and genetic variants. When observing the *NF1* mutational spectrum, one variant (c.1013A>G (p.Asp338Gly/p.?) was identified in three patients with constipation of which two patients were related. The variant c.2970_2972delAAT (p.Met992del) associated with a mild NF1 phenotype was identified in two related patients with constipation. This study is the first to explore the association between symptoms of constipation, NF1 severity, and *NF1* mutational spectrum. The results suggest an association between constipation and a high degree of illness burden. Awareness of this association among physicians could lead to more patients with NF1 being diagnosed with constipation. Constipation impacts on quality of life, hence a timely diagnosis and treatment will improve quality of life.

## Introduction

Neurofibromatosis type 1 (NF1) is a genetic disease with a complete penetrance and a variable expressivity^1^. NF1 is inherited in an autosomal dominant mode and caused by mutations in the *neurofibromin* gene *NF1* on chromosome 17q11.2^2^. Fifty percent of NF1 cases are inherited from a parent and the remaining cases are caused by de novo* NF1* variants^3^. With a worldwide birth incidence of 1:2000 to 1:3000, NF1 is a rather common rare disease^4,5^. The diagnosis can be established clinically with diagnostic features developing during childhood. Approximately 97% of cases have been diagnosed at the age of 11 years^6^. The most recognizable features of NF1 are café-au-lait macules, axillary and inguinal freckling and cutaneous and plexiform neurofibromas^7^. More severe NF1 complications are optic glioma, epilepsy, scoliosis, and plexiform neurofibromas with a lifelong risk of development of malignant peripheral nerve sheat tumor^7^. Apart from the physical features, common findings in patients with NF1 include minor mental impairment, learning disabilities and behavioural symptoms^7,8^. To date, only few NF1 genotype–phenotype correlations have been established. Approximately five percent of patients with NF1 have a large constitutional *NF1* deletion, the NF1 microdeletion syndrome that causes a relatively severe phenotype^9^. The 3-bp in frame deletion (c.2970-2972delAAT, p.Met992del) gives rise to a mild NF1 phenotype, estimated to occur in one percent of patients with NF1^10,11^.

Studies on gastrointestinal symptoms in patients with NF1 are few. A review on 24 case reports on gastrointestinal involvement in children with NF1 described dyspepsia, abdominal pain and distension, bowel obstruction, bleeding and constipation^12^. In a previous study we found that children with NF1 had a high prevalence of constipation (30%) compared to healthy controls and a higher number than expected had prolonged colonic transit time (19%) when compared to the literature^13^. Furthermore, we have recently reported that children, adolescents, and adults with NF1 had a significantly higher frequency of gastrointestinal symptoms relating to constipation compared to unaffected relatives; the adult odds ratio was 3.80; 95% CI 1.27–11.31, and the children/adolescent odds ratio was 6.4; 95% CI 1.45–28.24^14,15^. In the present study, we aimed to investigate the association between NF1 disease severity and the frequency of constipation in patients with NF1. Furthermore, we explored potential associations between constipation and the *NF1* mutational spectrum.

## Methods

### Participants

Patients were all recruited from the outpatient clinic at the Centre for Rare Diseases, Department of Paediatrics and Adolescent Medicine, Aarhus University Hospital in Denmark and all the participants had a clinical diagnosis of NF1 in accordance with by the National Institutes of Health Consensus Conference^16^.

The study population comprised 277 patients with NF1 of which 102 were between 4 and 17 years old [median age 10.3 years (interquartile range (IQR) 6.5)] and 175 were adults [median age 34.2 years (IQR 20.6)]. The 277 patients came from 207 unrelated families. They participated in a questionnaire study on gastrointestinal symptoms based on the validated Rome III diagnostic questionnaires developed for patients ≥ 4 years of age^17^. Cases were defined as NF1 patients with symptoms correlating to constipation (n = 49, 18%, of which 23 ≤ 18 years old) and controls were defined as NF1 patients without constipation (n = 228, 82%). A three self-labelling item questionnaire on NF1 was issued at the same time as the Rome III diagnostic questionnaire (a) at the patient’s regular follow-up at Centre for Rare Diseases or (b) by letter to the patients who were not scheduled for appointments at Centre for Rare Diseases during the recruitment period. The questionnaire and study population have previously been described in detail^14,15^.

### Protocol

The present study was based on (1) the results from the Rome III diagnostic questionnaire studies (constipation; yes or no); (2) a patient-administered questionnaire of three self-labelling items on self-perceived perception of NF1 illness burden, NF1 severity grade, and NF1 visibility grade; (3) an assessment of NF1 disease severity and visibility severity grade made by a physician; and (4) a *NF1* mutational analysis.

Study data were collected and managed using the Research Electronic Data Capture (REDCap) tool hosted at Department of Clinical Medicine at Aarhus University. REDCap is a secure, web-based application designed to support data capture for research^18^.

The participants and parents in cases with underaged patients, were introduced to the confidentiality procedures by the first author. Informed consent was obtained from participants and parents. Personally identifiable data have been omitted from the manuscript. The study complies with the Declaration of Helsinki and was approved by the Danish Data Protection Agency (ID: 1-16-02-271-15 & 1-16-02-501-15) and the Danish National Committee on Health Research Ethics (ID: 1-10-72-262-15).

### Data and analyses

#### Questionnaire

The self-labelling three-item questionnaire on self-perceived perception of NF1 was assessed using the items ‘How many symptoms of NF1 do you have?’ (many, some, few, very few or none) equal to “Perception of symptoms” and ‘How visible is NF1 when you are fully dressed and participate in a conversation?’ (very, some, almost not or not) equal to “Perception of visibility”, and ‘How bothered are you by NF1?’ (very, some, little, not) equal to “Perception of illness burden”. The assessment was subjective with no special conditions including the presence of constipation included. NF1 disease severity and visibility severity were assessed by the patient’s physician according to the Riccardi and Ablon scales^19,20^, see online appendix.

#### *NF1* mutational analysis on participants

Information on any result from previously performed *NF1* mutational analysis was collected by a review of medical records. If prior *NF1* mutational analysis had been undertaken, an analysis of NF1 cases and NF1 controls was performed at Department of Molecular Medicine, Aarhus University Hospital. Mutational analyses were carried out by Next Generation Sequencing, Sanger sequencing, and Multiplex Ligation-dependent Probe Amplification. Mutations were annotated using Human Genome Variation Society nomenclature, NM_000267.3. The interpretation of the pathogenicity of the novel mutations was based on the guidelines by American College of Medical Genetics and Genomics as described by Richards et al.^21^.

### Statistics

Statistical analyses were performed using the Stata 12 software (StataCorp, College Station, TX). The median and interquartile range of the age were reported in each group and the groups were compared using a permutation test for Mann–Whitney correction for continuous variables. Gender was reported as frequencies and gender distribution between cases and controls was compared using chi-square test. The distribution of genetic variants was reported as frequencies and cases and controls were compared using Fisher’s exact test. A *p* < 0.05 was considered statistically significant. Five simple logistic regression analyses followed by two multiple logistic regression analyses reporting two different regressions (model A and B) were performed with constipation as the dependent variable.

Model A investigated associations between constipation in NF1 and the independent variables disease severity and visibility severity scored by a physician. To ensure adequate numbers in each group in model A, disease severity was divided into minimal/mild and moderate/severe; Visibility severity was divided into mild/moderate and severe.

Model B investigated associations between constipation in NF1 and the independent variables self-perceived perception of NF1 illness burden, NF1 visibility and NF1 symptoms. To ensure adequate numbers in each group in model B; NF1 illness burden was divided into very/some and little/not; NF1 visibility was divided into very/some and almost not/or not; NF1 symptoms were divided into many/some, few/very few or none. ORs were reported along with 95% confidence intervals. Participants from the same family were assumed to be more homogeneous than those from different families and hence the between-family variation was considered in the logistic regression model. The variants of the mutational spectrum were reported as frequencies and the groups were compared using Fisher’s exact test. A *p* < 0.05 was considered statistically significant.

### Ethics approval and consent to participate

The study complies with the Declaration of Helsinki and was approved by the Danish Data Protection Agency (ID: 1-16-02-271-15 & 1-16-02-501-15) and the Ethics Committee of Denmark (ID: 1-10-72-262-15). Informed consent was obtained from patients and in the case of underaged patients from parents.

### Consent for publication

Not applicable.

## Results

### Association between NF1 disease and constipation

We observed no significant difference in age and gender between cases and controls; cases were 18.9 years (IQR: 23.6) and controls 25.0 years (IQR: 27), *p* = 0.217); 30 cases were females (61.2%) and 26 controls were females (55.3%), *p* = 0.445). Table 1 presents the results on NF1 disease severity and visibility severity and Table 2 presents self-perceived perceptions of NF1 among cases and controls. There was no statistically significant difference between the groups. The highest constipation rate was found among the patients who reported a high NF1 illness burden, Table 2. Hence, a sub-analysis was performed comparing only the cases and controls “not bothered” to “very bothered”. Cases had a significantly higher NF1 illness burden than controls when performing a sub-analysis comparing those “not bothered” (51 controls versus 3 cases) to those “very bothered” (22 controls versus 7 cases), (*p* = 0.013).

**Table 1:** Disease severity and visibility severity in 275 patients scored by a physician.

Variable	Patients with constipation* (Cases)	Patients without constipation* (Controls)	*p* value
**NF1 disease severity (Riccardi’s score)**			
Minimal/mild	32 (67%)	134 (59%)	0.326
Moderate/severe	16 (33%)	93 (41%)	
**NF1 visibility severity (Ablon’s score)**			
Mild/moderate	34 (71%)	144 (63%)	0.330
Severe	14 (29%)	83 (37%)	

*Data missing in one patient in each group.

**Table 2:** Self-perceived perception of NF1 in 276 patients.

Variable	Patients with constipation (cases)	Patients without constipation* (controls)	*p* value
**How many symptoms of NF1 do you have?**			
Few/very few or none	26 (53%)	139 (61%)	0.290
Many/some	23 (47%)	88 (39%)	
**How visible is NF1 when you are fully dressed and participate in a conversation?**			
Almost not or not	27 (55%)	139 (61%)	0.427
Very/some	22 (45%)	88 (39%)	
**How bothered are you by NF1?**			
Little/not	27 (55%)	157 (69%)	0.058
Very/some	22 (45%)	70 (31%)	
**Subanalysis on how bothered**			
Not	3 (30%)	51 (70%)	0.013
Very	7 (70%)	22 (30%)	

*Data missing in one patient.

There was no statistically significant effect neither in model A (assessment of NF1 disease severity and visibility severity by a physician) nor in model B (self-perceived perception of NF1 severity of symptoms, NF1 severity of visibility and of NF1 illness burden) and the frequency of having constipation; regression analyses are presented in Table 3.

**Table 3:** Simple logistic regression analysis at individual level with constipation as dependent variable.

Variable	Model A: Physician’s score	Model B: Self-perceived
	Odds ratio	Odds ratio
**Model A**		
Disease severity	0.72 (CI 0.38–1.28)	
Visibility severity	0.71 (CI 0.36–1.41)	
**Model B**		
Perception of symptoms		1.40 (CI 0.74–2.62)
Perception of visibility		1.29 (CI 0.67–2.44)
Perception of illness burden		1.83 (CI 0.95–3.52)
*Multiple logistic regression analysis with constipation as dependent variable reporting two different regressions*		
**Model A**		
Disease severity	0.77 (CI 0.39–1.53)	
Visibility severity	0.77 (CI 0.37–1.58)	
**Model B**		
Perception of symptoms		1.09 (CI 0.51–2.33)
Perception of visibility		1.04 (CI 0.48–2.24)
Perception of illness burden		1.74 (CI 0.79–3.80)

*CI* 95% Confidence interval.

### *NF1* mutational spectrum

In one case and 13 controls, no genetic variant was obtained due to fear of needles or no response to the mutational analyses invitation. Hence, information on *NF1* variants was obtained in 95% of all cases and controls. Seven classes of variants were considered: intragenic (missense, nonsense, frameshift, and splicing), deletion/duplication of exon(s), total gene deletion, and synonymous substitution. The proportion of all intragenic variants was close to identical: 94% among cases and 91% among controls. There was a higher frequency of the missense variants within the case group (23%) compared to the control group (10%). The reverse was observed for nonsense variants (8% vs. 23%). Hence, the overall proportion of all seven classes of variants differed significantly between the two groups (*p* = 0.004). Though, no associations were found between the variant classes and self-perceived perception of NF1 symptoms, visibility, and illness burden or NF1 disease severity and visibility severity scored by a physician (data not shown) between the two groups.

On exon level, the identified variants were evenly distributed within the *NF1* gene with no difference between cases and controls (*p* = 0.546). Evaluating potential exon hotspots by an accumulation of three or more variants present in one exon among cases, four exons^9,14,22^ had an accumulation of variants.

Among the cases, 42 different variants were identified. The likely pathogenic (Class 4) splice variant c.1013A>G (p.Asp338Gly/p?) was identified in two unrelated families. These families consisted of two and four members carrying the splice variant of which one and two, respectively, had constipation. Furthermore, in three families, two family members had constipation. The variants identified in these four families were c.1634C>A [p. (Ala545Glu)], c.2970_2972delAAT (p.Met992del) and c.5489C>T [p.(Pro1830Leu)]. The variant c.6792C>A (p.Ala2256_Lys2286del/r.6757_6858del) was identified in two unrelated patients with constipation.

## Discussion

In summary, we found that the strongest associated factor to constipation was the patient’s perception of illness burden. Constipation impacts on quality of life^22^ and could potentially increase the illness burden. Illness burden covered how bothered a patient was by NF1 and this study did not give information on how the patients experienced the NF1 illness burden. Previously, we have found that children with NF1 had prolonged colonic transit time when compared to the literature^13^. Abnormalities within the enteric, the peripheral or the central nervous system could cause chronic constipation. Further studies are needed to determine how constipation in patients with NF1 is associated with altered gastrointestinal motility. In addition, future investigations should focus on whether patients with constipation in general experience symptoms connected to the autonomous nervous system, e.g. urinary problems, dizziness, altered body temperature, or altered sensation/pain.

No clear association was seen between the frequency of constipation as an outcome and the following variables: NF1 phenotype severity estimated by physician scores on Ablon and Riccardi scales; self-perceived perception of NF1 symptom severity and visibility severity.

When observing the *NF1* mutational spectrum, one of the variants was identified in three of the patients with constipation representing two families; the splice variant in the 5′ tertile c.1013A>G (p.Asp338Gly/p.?) of exon 9. Interestingly, the variant c.2970_2972delAAT (p.Met992del) associated with the well described mild NF1 phenotype^10^ was identified in a family including two members with constipation. No association between neither symptoms of constipation between the classes of variants nor the location of variants on exon level was found. Further studies comprising functional analyses and search for modifier genes may clarify new potential pathogenesis mechanisms associated with constipation in NF1.

The strengths of our study are that all patients received the NF1 diagnosis by a specialist physician at the Centre of Rare Diseases; that the assessment of NF1 severity score was performed at the patient’s attendance; and in addition the majority (95%) of patients had a known NF1 genotype. All patients with proven or suspected NF1 in children, adolescents as well as adults in the western part of Denmark (comprising eight secondary and two tertiary hospitals) are referred to the Centre of Rare Diseases at Aarhus University Hospital, one of two Danish national centers of expertise in NF1. Thus, we believe that our population is representative of the total Danish NF1 population. Additionally, access to the Danish health system is free of charge, which means that patients from all socio-economic groups representing all degrees of NF1 severity are followed at our center. The latter reduces the risk of selection bias, though one must take into account that very mildly affected patients with NF1 may not wish to be followed at the center or could still remain undiagnosed.

The study has some limitations. We only evaluated NF1 severity and self-perception of NF1, hereby missing information on clinical characteristics. Though the study did not show a clear association between constipation and disease severity, it could be of interest to investigate the association between specific NF1 phenotype traits and the frequency of constipation particularly in the situation of variants occurring among more than one patient with constipation. In general, it is difficult to show associations between patient-reported gastrointestinal symptoms and the findings on motility investigations, e.g. investigation of gastrointestinal dysmotility in gastroparesis, diabetes mellitus and multiple upper and lower gastrointestinal symptoms^23–25^.

## Conclusion

In conclusion, this study is the first to explore the association between NF1 severity, symptoms of constipation and the *NF1* mutational spectrum. The results suggest an association between constipation and a high illness burden. This may result in a clinical benefit since awareness by physicians of the association between constipation and a perception of a high illness burden could lead to more patients with NF1 being diagnosed with constipation. Constipation impacts on quality of life^22^, and timely diagnosis and treatment will thus improve quality of life.

## Supplementary Information

Supplementary Information

## Data Availability

The datasets used and/or analysed during the current study are available from the corresponding author on reasonable request.

## Abbreviations

NF1: Neurofibromatosis type 1
REDCap: Research electronic data capture
