# Supplementary material for: Exploring associations between constipation, severity of neurofibromatosis type 1 and NF1 mutational spectrum
Source: Sci Rep. 2021 Apr 28;11:9179. doi: 10.1038/s41598-021-87686-x (PMC8080678; doi:10.1038/s41598-021-87686-x)
Supplement: Supplementary file 1 — Supplementary Information [file 41598_2021_87686_MOESM1_ESM.docx]

## Online Appendix

| *The Riccardi Scale^1^ – NF1 disease severity* | |
| --- | --- |
| Minimal | The presence of few features of NF with no compromise of health or well-being |
| Mild | The presence of enough stigmata to make the disease obvious and a source of concern, but without significant compromise of health. For example, the patient may exhibit facial café au lait spots or a modest number of cutaneous or deep neurofibromas. |
| Moderate | An unequivocal compromise of health and well-being, but the compromise can be reasonably well managed, is not intractable, and will not invariably lead to a shortened life span. Because many features of NF can lead to this level of clinical problems, the category of severity grade 3 is necessarily broad, spanning a relatively large age range. |
| Severe | The presence of serious compromise that is intractable, is managed or treated only with difficulty, or is associated, at least statistically, with a shortened life span. Mental retardation, drug-resistant seizures, brain tumours, and malignant tumours contribute to this category of severity. |
| *The Ablon Scale^2^ – NF1 visibility severity* | |
| Mild | Essentially no tumours are visible outside of normal Western clothing areas; gait and posture appear unremarkable when casually observed |
| Moderate | Some tumours appear on the neck, face, and hands, and mild scoliosis or other skeletal features are present but without a noticeable limp. |
| Severe | Numerous tumours appear on the face; optic glioma (tumour) affects sight and the eye socket; severe scoliosis or skeletal features are present causing a noticeable limp. |

^1^ Adapted from; Riccardi VM, Kleiner B. Neurofibromatosis: a neoplastic birth defect with two age peaks of severe problems. Birth Defects Orig Artic Ser 1977;13(3C):131-138. ^2^ Adapted from; Ablon J. Gender response to neurofibromatosis 1. Soc Sci Med 1996 Jan;42(1):99-109.
